# Ovarian Cyst Fluid of Serous Ovarian Tumors Contains Large Quantities of the Brain Amino Acid N-acetylaspartate

**DOI:** 10.1371/journal.pone.0010293

**Published:** 2010-04-22

**Authors:** Eva Kolwijck, Ron A. Wevers, Udo F. Engelke, Jannes Woudenberg, Johan Bulten, Henk J. Blom, Leon F. A. G. Massuger

**Affiliations:** 1 Department of Obstetrics and Gynecology, Radboud University Nijmegen Medical Centre, Nijmegen, The Netherlands; 2 Laboratory of Pediatrics and Neurology, Radboud University Nijmegen Medical Centre, Nijmegen, The Netherlands; 3 Department of Gastroenterology, Radboud University Nijmegen Medical Centre, Nijmegen, The Netherlands; 4 Department of Pathology, Radboud University Nijmegen Medical Centre, Nijmegen, The Netherlands; 5 Department of Clinical Chemistry, VU University Medical Center, Amsterdam, The Netherlands; University of Southampton, United Kingdom

## Abstract

**Background:**

In humans, N-acetyl L-aspartate (NAA) has not been detected in other tissues than the brain. The physiological function of NAA is yet undefined. Recently, it has been suggested that NAA may function as a molecular water pump, responsible for the removal of large amounts of water from the human brain. Ovarian tumors typically present as large cystic masses with considerable fluid accumulation.

**Methodology and Principal Findings:**

Using Gas Chromatography-Mass Spectrometry, we demonstrated that NAA was present in a high micromolar concentration in oCF of epithelial ovarian tumors (EOTs) of serous histology, sometimes in the same range as found in the extracellular space of the human brain. In contrast, oCF of EOTs with a mucinous, endometrioid and clear cell histological subtype contained a low micromolar concentration of NAA. Serous EOTs have a cellular differentiation pattern which resembles the lining of the fallopian tube and differs from the other histological subtypes. The NAA concentration in two samples of fluid accumulation in the fallopian tube (hydrosalpinx) was in the same ranges as NAA found in oCF of serous EOTs. The NAA concentration in oCF of patients with serous EOTs was mostly 10 to 50 fold higher than their normal serum NAA concentration, whereas in patients with other EOT subtypes, serum and cyst fluid NAA concentration was comparable.

**Conclusions and Significance:**

The high concentration of NAA in cyst fluid of serous EOTs and low serum concentrations of NAA in these patients, suggest a local production of NAA in serous EOTs. Our findings provide the first identification of NAA concentrations high enough to suggest local production outside the human brain. Our findings contribute to the ongoing research understanding the physiological function of NAA in the human body.

## Introduction

N-acetyl L-aspartate (NAA) is the second most abundant free amino acid in the human brain.[1] Neuronal cells contain 20×10^3^ µmol/L NAA, whereas in the extracellular space of the brain, the NAA concentration ranges between 80 and 100 µmol/L.[1] In spite of these high amounts of NAA, its metabolic and neurochemical functions remain controversial. NAA is thought to function as an important intracellular osmolyte and serve as a source of acetate for lipid and myelin synthesis in glia cells. Furthermore, NAA is considered to be an intermediate in the formation of the neuropeptide N-acetylaspartylgutamate, a storage vehicle for neuronal aspartate and glutamate [1]–[3] Interestingly, a patient has been described with a biosynthesis defect of NAA. This patient had a severe neurological disorder with delayed myelination but was still alive at 8 years and 6 months of age.[4]

Recently, it has been proposed that the NAA system functions as a molecular water pump (MWP) operating between neurons and oligodendrocytes.[5] In contrast to osmolytic transport, MWPs are entities that actively use intercompartmental cotransport of water against a gradient.[6] Following this hypothesis, NAA is thought to be primarily responsible for the active removal of metabolic water from myelinated neurons, of which the membranes are known to have a very low water permeability.[6]

Concentrations of NAA in body fluid outside the brain are very low. In CSF, plasma, and amniotic fluid of healthy individuals, mean ± SD (range) concentrations of NAA have been reported to be 1.51±0.89 (0.25–2.83) µmol/L, 0.44±0.20 (0.17–0.84) µmol/L, and 1.27±0.74 (0.30–2.55) µmol/L, respectively.[7] Considerable amounts of extra-neuronal NAA have been found in the lens of the eye and in peritoneal mast cells, although this has never been confirmed for humans.[8], [9] Using magnetic resonance spectroscopy, NAA was shown in detectable amounts in human ovarian cyst fluid (oCF).[10], [11]

Epithelial ovarian tumors (EOTs) are extremely heterogeneous entities which are mostly filled with large amounts of oCF.[12] The aim of the present study was to further investigate the presence of NAA in oCF from patients with EOTs of different histological subtypes. As the mechanism of cyst formation of EOTs is still unknown, this study was conducted to better understand the possible role of NAA in body water management.

## Results

### NAA in cyst fluid of epithelial ovarian tumors

**Figure 1:**
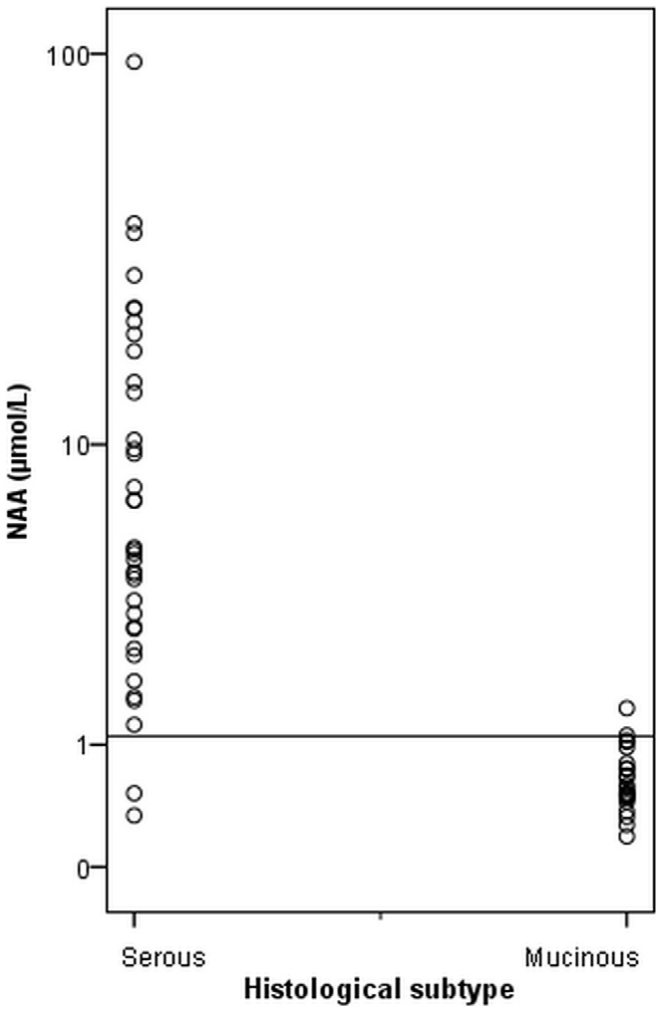
NAA (µmol/L) concentration of individual patients with serous (n = 36) and mucinous (n = 23) tumors. Values are presented by dots on a logarithmic scale. A horizontal line represents the cut-off value of 1.1 µmol/L NAA.

The median (25^th^–75^th^ percentile) NAA concentration in oCF of serous, mucinous, endometrioid and clear cell EOTs was 5.1 (2.9–17.0), 0.6 (0.5–0.8), 1.2 (0.8–4.5), and 1.3 µmol/L, respectively. NAA in serous EOTs was significantly higher compared to the other histological subtypes (p<0.001, Kruskal Wallis test). The NAA concentration in one third (n = 12) of the serous EOTs was more than 10.0 µmol/L. Serous (n = 36) and mucinous (n = 23) EOTs could almost perfectly be distinguished by a cut-off value of 1.1 µmol/L (Figure 1). All, except one, oCF samples of mucinous tumors contained less than 1.1 µmol/L NAA, whereas all, except two, oCF samples of serous tumors contained more than 1.1 µmol/L NAA (p<0.001, Mann-U Whitney test, Figure 1).

### NAA in cyst fluid of epithelial ovarian tumors of serous histology

In the group of serous EOTs (n = 36), median (25^th^–75^th^ percentile) NAA concentration was 4.9 (3.8–7.3), 1.6 (0.5–14.7), and 8.2 (2.9–20.7) µmol/L for malignant (n = 9), borderline (n = 3) and benign (n = 24) tumors, respectively. No significant differences were found between the groups (p = 0.343, Kruskal-Wallis test). In addition, no significant correlation was found between the largest diameter of the serous tumors and the NAA concentration (p = 0.246, Pearson's correlation test, data not shown).

### NAA in cyst fluid of malignant epithelial ovarian tumors

Table 1 summarizes the data of patients with epithelial ovarian cancer (n = 25). Endometrioid carcinomas (n = 8) showed the largest variation in NAA concentration, ranging from 0.07 to 11.8 µmol/L (Table 1). Of the endometrioid carcinomas, 4 samples showed an NAA concentration below the arbitrary cut-off 1.1 µmol/L. Table 1 also lists the NAA concentration of serous (n = 9, range: 1.2–22.8 µmol/L), mucinous (n = 6, range: 0.37–0.80 µmol/L) and clear cell (n = 2, range: 0.07 and 2.5 µmol/L) ovarian carcinomas. When all carcinomas, regardless of subtype, were grouped by FIGO stage, NAA concentration in oCF from patients with advanced stage disease (stage III and IV, n = 14, median: 4.2, 25^th^–75^th^ percentile: 1.1–7.2 µmol/L) was significantly higher than NAA concentration in oCF from patients with early stage disease (stage I and II, n = 11, median: 0.8, 25^th^–75^th^ percentile: 0.5–1.5 µmol/L; p = 0.009, Mann U Whitney test). No correlation was found between tumor size and NAA concentration (p = 0.612, Pearson's correlation test).

**Table 1:** NAA (µmol/L) in oCF of patients with serous (n = 9), mucinous (n = 6), endometrioid (n = 8) and clear cell (n = 2) ovarian cancer.

Patient no.	Histopathological diagnosis	FIGO stage	Tumor size (cm)	NAA (µmol/L)
1.	Serous cystadenocarcinoma	IIIc	8	1.2
2.	Serous cystadenocarcinoma	IIIb	9	4.3
3.	Serous cystadenocarcinoma	IIIc	9	3.5
4.	Serous cystadenocarcinoma	IIIc	9	5.1
5.	Serous cystadenocarcinoma	IV	9	4.1
6.	Serous cystadenocarcinoma	IV	9	7.6
7.	Serous cystadenocarcinoma	IIIb	12	4.9
8.	Serous cystadenocarcinoma	IIIc	18	22.8
9.	Serous cystadenocarcinoma	IIIc	21	7.0
10.	Mucinous cystadenocarcinoma	IIIb	10	0.68
11.	Mucinous cystadenocarcinoma	IIIb	13	0.80
12.	Mucinous cystadenocarcinoma	Ic	17	0.37
13.	Mucinous cystadenocarcinoma	Ia	18	0.52
14.	Mucinous cystadenocarcinoma	IIIa	20	0.33
15.	Mucinous cystadenocarcinoma	Ic	26	0.50
16.	Endometrioid cystadenocarcinoma	IIb	4	0.8
17.	Endometrioid cystadenocarcinoma	Ic	9	1.5
18.	Endometrioid cystadenocarcinoma	IIb	14	0.9
19.	Endometrioid cystadenocarcinoma	Ia	18	5.4
20.	Endometrioid cystadenocarcinoma	Ia	22	0.9
21.	Endometrioid cystadenocarcinoma	IIb	22	1.6
22.	Endometrioid cystadenocarcinoma	IIIb	22	11.8
23.	Endometrioid cystadenocarcinoma	Ic	30	0.5
24.	Clear cell cystadenocarcinoma	IIIa	14	2.5
25.	Clear cell cystadenocarcinoma	Ia	23	0.1

### NAA concentration in ascites fluid and serum of patients with ovarian tumors

The NAA concentration in ascites and corresponding oCF is listed in Table 2. Ascites and oCF concentrations of NAA were comparable for each patient. Therefore, the concentration of NAA in ascites differed between patients with different histological subtypes of EOTs as well.

**Table 2:** NAA (µmol/L) in ovarian cyst fluid and ascites.

Patient no.	Histology	Subtype	NAA (µmol/L) cyst fluid	NAA (µmol/L) ascites
1.	Malignant	Serous	4.9	5.0
2.	Malignant	Serous	1.2	1.0
3.	Malignant	Serous	-	24.5
4.	Malignant	Mucinous	0.8	0.4
5.	Malignant	Clear cell	0.1	0.6

The NAA serum concentration ranged between 0.2 and 0.8 µmol/L for all patients with ovarian tumors (n = 7, data not shown), independent of the histological subtype of the tumor. These values are within the reported reference range.[7]

### NAA concentration in hepatic cyst fluid and hydrosalpingeal fluid

**Figure 2:**
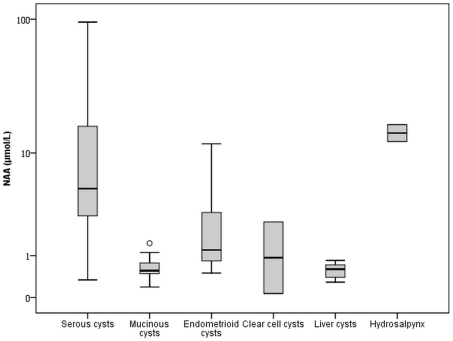
NAA (µmol/L) in cyst fluid of patients with serous (n = 36), mucinous (n = 23), endometrioid (n = 8) and clear cell (n = 2) EOT, patients with hepatic cysts (n = 10) and hydrosalpinges (n = 2), presented on a logarithmic scale.

Figure 2 illustrates the boxplots of NAA concentration in oCF of ovarian tumors grouped by histological subtype, in hepatic cyst fluid and in hydrosalpingeal fluid. The NAA concentration was very low in cyst fluid from patients with PCLD (n = 10). The median (25^th^–75^th^ percentile) concentration amounted to 0.6 (0.4–0.8) µmol/L and ranged between 0.3 and 0.9 µmol/L. The NAA concentration in fluid from the two hydrosalpinges amounted to 12.3 and 16.6 µmol/L. These values are in the same range as the NAA concentration in oCF of serous EOTs.

## Discussion

This study shows that NAA is present in low micromolar concentration in all ovarian cyst fluid samples. Serous ovarian tumors however, contained a significantly higher oCF concentration of NAA than mucinous, endometrioid and clear cell tumors. The median NAA concentration in serous EOTs was 5.1 µmol/L, whereas one third of all samples contained an NAA concentration above 10.0 µmol/L. The NAA concentration in serous EOTs was 5 to 50 fold higher than the NAA concentration previously found in CSF, serum and amniotic fluid of healthy individuals.[7] Some serous EOTs contained an NAA oCF concentration in the same range as found in the extracellular space of the human brain and all serous tumors by far exceeded the NAA concentration in human serum.[1] Interestingly, the oCF NAA concentration in serous EOTs did not differ between benign, borderline and malignant histology, indicating that NAA expression might be specific for EOTs with a serous cell differentiation.

NAA was present in fluid of mucinous EOTs and hepatic cysts as well but this concentration was comparable to the NAA concentration in human serum.[7] Serous and mucinous EOTs could almost perfectly be distinguished when using an NAA cut-off value of 1.1 µmol/L. Most endometrioid and clear cell carcinomas contained an NAA concentration which was comparable to the concentration of NAA in human serum or CFS. However, in some of these samples a higher NAA concentration was found. This may be explained by the common presence of cells of different ovarian cancer subtypes within a single tumor. In general, approximately 6% of the ovarian carcinomas can be classified as mixed type ovarian carcinomas (at least two histologically distinctive elements are present in at least 10% of the tumor surface).[13] However, more frequently, ovarian carcinomas are found to exist of a main subtype and a small percentage of cells representing a different histology.[13] Our findings indicate that the brain amino acid NAA is predominantly present in oCF of serous EOTs and therefore, seems to be characteristic for this specific histological subtype. Therefore, NAA analysis in oCF might be used to diagnostically distinguish between serous EOTs and other EOT subtypes.

Although 90% of the ovarian tumors derive from the epithelial surface of the ovary, EOTs are not a single disease entity, but instead comprise a heterogeneous group of tumors.[14], [15] These tumors are classified according to their pattern of histological differentiation.[16] Serous tumors bear strong resemblance to the normal cells lining the fallopian tube. Mucinous tumors show strong similarity to epithelium of the intestine and endometrioid and clear cell tumors are morphologically identical to cells of the lining of the endometrium.[17] Interestingly, the normal cellular lining of the ovary does not resemble any of these organs. The high concentration of NAA found in oCF of serous EOT, independent of their malignant potential, might be due to their specific cellular differentiation pattern, which resembles the lining of the fallopian tube and differs from the other histological subtypes. This finding is supported by our observation of similar high amounts of NAA found in hydrosalpingeal fluid, which is a fluid accumulation in the fallopian tube due to distal obstruction.[12] However, the question remains why NAA is found in such high amounts in oCF of serous EOT as its presence and function have been specifically been assigned to the human brain.

So far, the brain is the only organ where NAA synthesis has been demonstrated in humans. However, two animal studies have revealed extra neuronal NAA synthesis.[8], [9] In the first study, NAA has been demonstrated in ocular fluid, the lens and retina of fish and mammals.[9] Despite a low micromolar serum concentration of NAA, high amounts of NAA and all components required for synthesis of NAA were present in the eye of these animals. In the present study, the NAA concentration in all serum samples was within the normal range of 0.17–0.84 µmol/L [7] and did not vary between patients with different histological subtypes. The NAA concentration in oCF of serous tumors was mostly 10 to 50 fold higher than the normal serum NAA concentration. As serum and oCF samples were simultaneously taken, our data suggest a local NAA synthesis in the tumor or a pump mechanism which actively pumps NAA from the serum into the oCF. In other ovarian tumor subtypes, serum and cyst fluid concentration of NAA was comparable, which might suggest that local NAA production primarily occurs in EOTs of serous histology. Peripheral synthesis of NAA also has been described for peritoneal mast cells of the rat.[8] Release of NAA from mast cells was found to occur rapidly when degranulating agents were used.[8] Although this finding has not yet been confirmed in humans, release of NAA from mast cells might be an alternative explanation for the high NAA concentration in oCF and ascites. It has been demonstrated that mast cells are frequently present in stroma tissue of several cancer types, including ovarian carcinomas.[18], [19] A growing number of studies has shown that the mast cell count correlates with tumor stage, tumor invasiveness and prognosis.[18] In addition, mast cells are known to be present in ascites of cancer patients as well.[20] However, we found that a high concentration of NAA in oCF and ascites was characteristic for patients with serous EOTs, independent of the malignant potential of the tumor. Mast cells in ovarian stromal tissue have been demonstrated in all histological subtypes of EOTs and numbers were found to be increased in carcinomas compared to their normal tissue counterparts.[19], [21] Therefore, we consider a local NAA production in serous EOTs as the most likely explanation for the higher concentration of NAA in serous oCF samples.

Recently, Baslow proposed that the NAA cycle itself might serve as a neuronal mechanism to remove large amounts of water generated during action potential development.[1], [5] To prevent swelling of neurons, this metabolic water has to be eliminated, which has been to occur energy driven and against a water gradient. If the NAA system would function as a so called molecular water pump, as a result of the enzymatic hydrolysis of NAA in the oligodendrocytes, its obligated water can be released. This would subsequently result in a hypoosmotic extracellular space, from which it can be removed from the brain.[5] Canavan Disease (CD) is a genetic disorder which causes NAA accumulation in the brain due to a deficiency in the NAA-degrading enzyme aspartoacylase.[2] The disease is characterized by the buildup of excessive fluid within the myelin lamellae, in swollen astrocytes and in extracellular fluid vacuoles.[5] Baslow suggests that this accumulation of NAA is responsible for the macrocephaly in CD.

EOTs, benign as well as malignant, mostly appear as large cystic masses, sometimes diagnosed with a diameter up to 50 cm. In mucinous tumors, cysts are thought to be formed by mucus producing epithelial cells. Mucus is known to contain large amounts of glycoprotein with a high carbohydrate content.[22] In contrast, serous EOT are usually filled with a clear and watery fluid,[12] but the mechanism responsible for serous cyst formation is still unknown. On the basis of our findings and taking into account the proposed role of NAA in body water management [1], [5], we hypothesize that high amounts of NAA in oCF of serous EOTs may be related to accumulation of water in the tumor and may contribute to cyst formation. In addition, this proposed mechanism might also be involved in the formation of ascites in patients with serous adenocarcinomas and in accumulation of hydrosalpingeal fluid. However, as yet there is no confirmatory study in literature to prove the role of NAA in water management. Therefore, our findings might contribute to the understanding of the so far undefined function of NAA. More evidence is required for the putative role of serous epithelial cells in NAA synthesis and cyst formation.

## Methods

### Ethics statement

The ovarian cyst fluid (oCF) samples involved in this study were obtained after surgical removal of the ovarian tumor. The hepatic and hydrosalpingeal biomaterial and ascites involved in this study were collected through a clinical (therapeutic) procedure. The informed consent for these procedures was obtained verbally in presence of a witness and documented in the patient's medical record. As part of the procedure we asked the patient for permission to use the residual biomedical waste (i.e. cyst fluid) for research purposes. The Institutional Review Board from the Radboud University Nijmegen Medical Center (RUNMC) considers that the use of residual biomedical waste presents no more than minimal risk or harm to the participants and involves no procedures for which written consent is normally required outside of the research context. The study was approved by the ethical board of the RUNMC (file number AMO 09/107).

### Patients and ovarian cyst fluid

Ovarian cyst fluid (oCF) was retrieved from our Radboud University Nijmegen Medical Center (RUNMC) biobank. This biobank contains samples of patients who underwent primary surgery for with an ovarian tumor at the RUNMC in the period between 1998 and 2008. Samples were collected by aseptic fine needle aspiration at the Department of Pathology immediately after surgical removal of the ovarian tumor. After cooled transport to the laboratory, all samples were centrifuged at 3000×g for 10 minutes and the supernatant was stored at −35°C in small portions until use. For the purpose of this study, 80 oCF samples were randomly selected from our biobank. Complete histopathological reports and slides of all patients were reviewed for correct histopathological diagnosis by one pathologist (JB), specialized in gynecological pathology. Eleven oCF samples were excluded because the ovarian tumor was non-epithelial or not primary ovarian-derived. Histopathological diagnosis of the remaining 69 EOTs revealed 36 serous, 23 mucinous, 8 endometrioid and 2 clear cell tumors. Of these 69 EOTs, 25 were malignant, 8 tumors were of borderline malignancy and 36 tumors were benign (Table 3). Of 7 patients with an EOT preoperative serum was collected as well. These samples were obtained from 3 patients with serous tumors (2 malignant and one borderline), 2 patients with mucinous tumors (malignant and borderline), a patient with an endometrioid carcinoma and a patient with a clear cell carcinoma. Of 5 patients, ascites samples were obtained during primary surgery. One ascites sample was obtained without the availability of an oCF sample. Informed consent was obtained from all participants.

**Table 3:** Overview of biological fluid samples grouped by origin.

		Histopathology		
Origin of biological fluid	n	Malignant	Borderline	Benign
**Ovarian cyst**	69	25	8	36
Serous subtype	36	9	3	24
Mucinous subtype	23	6	5	12
Endometrioid subtype	8	8	0	0
Clear cell subtype	2	2	0	0
**Hepatic cyst**	10	0	0	10
**Ascites**	5	5	0	0
**Hydrosalpinx**	2	0	0	2
**Serum**	7	5	2	0

### Hepatic cyst fluid

Cyst fluid from 45 patients with polycystic liver disease (PCLD) was obtained between 2002 and 2008 by percutaneous cyst aspiration or laparoscopic cyst fenestration and stored in aliquots at −20°C. For this study, we randomly selected hepatic cyst fluid samples of 10 patients. None of these patients had a previous diagnosis of carcinoma. The samples were centrifuged at 3000×g for 10 minutes.

### Hydrosalpingeal fluid

Hydrosalpingeal fluid accumulates in the Fallopian tube due to distal obstruction. This fluid was collected from 2 salpinges of 1 patient undergoing laparoscopic salpingectomy for presence of bilateral hydrosalpinges. Aspiration of the fluid was performed during surgery by fine needle aspiration before the fallopian tubes were excised. Verbal consent was obtained and documented in the patient's file. The samples were centrifuged at 3000×g for 10 minutes and the supernatant was stored at −35°C until use.

### N-acetyl-L-aspartic acid (NAA)

NAA (µmol/L) was measured using a modified stable isotope dilution Gas Chromatography-Mass Spectrometry (GC-MS) method essentially as described by Jakobs *et al.* [7] In short, 1 nmol [D3]-NAA was added as internal standard to 100 µl of CF. This was also added to aqueous standards. The samples were acidified with 30 µl HCL (6 mol/l) to pH <2 and saturated with NaCl. Thereafter, the sample was extracted four times with 2 ml of an ethylacetate-2-propanol mixture (v/v 10∶1). The collected organic fractions were dried by anhydrous Na_2_SO_4_ and subsequently evaporated to dryness at 40°C under a gentle stream of N_2_. NAA was converted to its di-isopropyl derivative by adding 500 µl isopropanol and 10 µl 6 mol/l HCl and subsequent heating for 1 hour at 120°C. An aliquot of 1 µl of this mixture was analysed by GC-MS. GC-MS analysis of 1 µl of this mixture was performed on an Agilent Technologies GC system 6890N with a 5973 MS detector. The gas chromatographic separation was achieved on a CPSil-88 capillary fused silica column (25 m×0.25 mm, df = 0.28 µm, Chrompack Int., Middelburg, The Netherlands). Helium was used as carrier gas. The oven temperature was kept at 80°C for 1 min and then raised to 240°C at a rate of 30°C/min. The interface and source temperatures were kept at 240°C. The inter-assay variation for this method was 8% (n = 5) and the intra-assay variation was 2% (n = 10). All samples were analysed within the linear range of the standard curve (0–20 nmol/L). If concentrations were above the standard curve, samples were diluted with water.

### Clinicopathological characteristics of patients with an EOT

Complete histopathological reports and slides of all patients were reviewed for correct histopathological diagnosis by one pathologist (JB), specialized in gynecological pathology. From the medical and pathological records of the patients with an EOTs, pathological diagnosis, histological subtype, largest tumor diameter (cm), and, in case of a malignancy, FIGO stage were scored. Staging was performed according to the criteria of the International Federation of Gynaecologists and Obstetricians (FIGO).[23]

### Statistical analysis

Statistical analyses were carried out using SPSS 16.0.2 software (SPSS Benelux BV, Gorinchem, the Netherlands). Values are shown as median with 25^th^–75^th^ percentile (µmol/L). Differences between two groups were tested by Mann U Whitney, and for more than 2 groups by Kruskal Wallis tests. Correlations between oCF NAA and ovarian tumor size were analyzed by Spearman's rank correlation testing. P values of <0.05 were regarded as statistically significant.
